# Supplementary material for: Unveiling the Bioactive Potential of the Invasive Jellyfish Phyllorhiza punctata Through Integrative Transcriptomic and Proteomic Analyses
Source: Biomolecules. 2025 Aug 4;15(8):1121. doi: 10.3390/biom15081121 (PMC12383608; doi:10.3390/biom15081121)
Supplement: Supplementary file 1 [file biomolecules-15-01121-s001.zip › Suplementary Material/FileS3/PEPFOLD4/XP_065067347_5268/clusters.html]

 PEP-FOLD clusters


Download archive of all models  here   

|  |  |  |
| --- | --- | --- |
| # | file | sOPEP |
| # cluster 1 - Size: 1 |  |  |
|  | XP\_065067347\_5268-model1 | -106.004 |
| # cluster 2 - Size: 2 |  |  |
|  | XP\_065067347\_5268-model2 | -103.707 |
|  | XP\_065067347\_5268-model2.1 | -101.139 |
| # cluster 3 - Size: 2 |  |  |
|  | XP\_065067347\_5268-model3 | -103.036 |
|  | XP\_065067347\_5268-model3.1 | -99.8084 |
| # cluster 4 - Size: 1 |  |  |
|  | XP\_065067347\_5268-model4 | -99.3121 |
| # cluster 5 - Size: 1 |  |  |
|  | XP\_065067347\_5268-model5 | -98.3581 |
| # cluster 6 - Size: 1 |  |  |
|  | XP\_065067347\_5268-model6 | -96.1369 |
| # cluster 7 - Size: 3 |  |  |
|  | XP\_065067347\_5268-model7 | -96.086 |
|  | XP\_065067347\_5268-model7.1 | -82.5852 |
|  | XP\_065067347\_5268-model7.2 | -81.8241 |
| # cluster 8 - Size: 1 |  |  |
|  | XP\_065067347\_5268-model8 | -95.0112 |
| # cluster 9 - Size: 1 |  |  |
|  | XP\_065067347\_5268-model9 | -94.2921 |
| # cluster 10 - Size: 2 |  |  |
|  | XP\_065067347\_5268-model10 | -94.0499 |
|  | XP\_065067347\_5268-model10.1 | -83.1827 |
| # cluster 11 - Size: 1 |  |  |
|  | XP\_065067347\_5268-model11 | -93.7724 |
| # cluster 12 - Size: 1 |  |  |
|  | XP\_065067347\_5268-model12 | -93.0976 |
| # cluster 13 - Size: 1 |  |  |
|  | XP\_065067347\_5268-model13 | -92.4453 |
| # cluster 14 - Size: 2 |  |  |
|  | XP\_065067347\_5268-model14 | -91.1165 |
|  | XP\_065067347\_5268-model14.1 | -87.0094 |
| # cluster 15 - Size: 1 |  |  |
|  | XP\_065067347\_5268-model15 | -90.0032 |
| # cluster 16 - Size: 1 |  |  |
|  | XP\_065067347\_5268-model16 | -89.0804 |
| # cluster 17 - Size: 2 |  |  |
|  | XP\_065067347\_5268-model17 | -89.0753 |
|  | XP\_065067347\_5268-model17.1 | -88.081 |
| # cluster 18 - Size: 1 |  |  |
|  | XP\_065067347\_5268-model18 | -88.7112 |
| # cluster 19 - Size: 1 |  |  |
|  | XP\_065067347\_5268-model19 | -86.4732 |
| # cluster 20 - Size: 1 |  |  |
|  | XP\_065067347\_5268-model20 | -85.7568 |
| # cluster 21 - Size: 1 |  |  |
|  | XP\_065067347\_5268-model21 | -85.6926 |
| # cluster 22 - Size: 1 |  |  |
|  | XP\_065067347\_5268-model22 | -85.6349 |
| # cluster 23 - Size: 1 |  |  |
|  | XP\_065067347\_5268-model23 | -85.3937 |
| # cluster 24 - Size: 1 |  |  |
|  | XP\_065067347\_5268-model24 | -85.3844 |
| # cluster 25 - Size: 1 |  |  |
|  | XP\_065067347\_5268-model25 | -84.0059 |
| # cluster 26 - Size: 1 |  |  |
|  | XP\_065067347\_5268-model26 | -83.8968 |
| # cluster 27 - Size: 1 |  |  |
|  | XP\_065067347\_5268-model27 | -83.8307 |
| # cluster 28 - Size: 1 |  |  |
|  | XP\_065067347\_5268-model28 | -83.754 |
| # cluster 29 - Size: 1 |  |  |
|  | XP\_065067347\_5268-model29 | -83.4489 |
| # cluster 30 - Size: 1 |  |  |
|  | XP\_065067347\_5268-model30 | -83.4179 |
| # cluster 31 - Size: 1 |  |  |
|  | XP\_065067347\_5268-model31 | -82.5177 |
| # cluster 32 - Size: 1 |  |  |
|  | XP\_065067347\_5268-model32 | -81.8218 |
| # cluster 33 - Size: 1 |  |  |
|  | XP\_065067347\_5268-model33 | -81.6074 |
| # cluster 34 - Size: 1 |  |  |
|  | XP\_065067347\_5268-model34 | -81.5161 |
| # cluster 35 - Size: 1 |  |  |
|  | XP\_065067347\_5268-model35 | -80.9364 |
| # cluster 36 - Size: 1 |  |  |
|  | XP\_065067347\_5268-model36 | -80.5603 |
| # cluster 37 - Size: 1 |  |  |
|  | XP\_065067347\_5268-model37 | -80.3412 |
| # cluster 38 - Size: 1 |  |  |
|  | XP\_065067347\_5268-model38 | -80.1847 |
| # cluster 39 - Size: 1 |  |  |
|  | XP\_065067347\_5268-model39 | -80.1633 |
| # cluster 40 - Size: 1 |  |  |
|  | XP\_065067347\_5268-model40 | -80.0085 |
| # cluster 41 - Size: 1 |  |  |
|  | XP\_065067347\_5268-model41 | -79.4786 |
| # cluster 42 - Size: 1 |  |  |
|  | XP\_065067347\_5268-model42 | -79.137 |
| # cluster 43 - Size: 1 |  |  |
|  | XP\_065067347\_5268-model43 | -79.0893 |
| # cluster 44 - Size: 1 |  |  |
|  | XP\_065067347\_5268-model44 | -77.9237 |
| # cluster 45 - Size: 1 |  |  |
|  | XP\_065067347\_5268-model45 | -77.4455 |
| # cluster 46 - Size: 1 |  |  |
|  | XP\_065067347\_5268-model46 | -76.5731 |
| # cluster 47 - Size: 2 |  |  |
|  | XP\_065067347\_5268-model47 | -76.176 |
|  | XP\_065067347\_5268-model47.1 | -75.6551 |
| # cluster 48 - Size: 1 |  |  |
|  | XP\_065067347\_5268-model48 | -75.8663 |
| # cluster 49 - Size: 1 |  |  |
|  | XP\_065067347\_5268-model49 | -75.7594 |
| # cluster 50 - Size: 1 |  |  |
|  | XP\_065067347\_5268-model50 | -75.6311 |
| # cluster 51 - Size: 1 |  |  |
|  | XP\_065067347\_5268-model51 | -75.5448 |
| # cluster 52 - Size: 1 |  |  |
|  | XP\_065067347\_5268-model52 | -75.5115 |
| # cluster 53 - Size: 1 |  |  |
|  | XP\_065067347\_5268-model53 | -75.2319 |
| # cluster 54 - Size: 1 |  |  |
|  | XP\_065067347\_5268-model54 | -74.7044 |
| # cluster 55 - Size: 1 |  |  |
|  | XP\_065067347\_5268-model55 | -74.5406 |
| # cluster 56 - Size: 1 |  |  |
|  | XP\_065067347\_5268-model56 | -74.4718 |
| # cluster 57 - Size: 1 |  |  |
|  | XP\_065067347\_5268-model57 | -73.8766 |
| # cluster 58 - Size: 1 |  |  |
|  | XP\_065067347\_5268-model58 | -73.3423 |
| # cluster 59 - Size: 1 |  |  |
|  | XP\_065067347\_5268-model59 | -73.3351 |
| # cluster 60 - Size: 1 |  |  |
|  | XP\_065067347\_5268-model60 | -73.1654 |
| # cluster 61 - Size: 1 |  |  |
|  | XP\_065067347\_5268-model61 | -73.1564 |
| # cluster 62 - Size: 1 |  |  |
|  | XP\_065067347\_5268-model62 | -72.5859 |
| # cluster 63 - Size: 1 |  |  |
|  | XP\_065067347\_5268-model63 | -72.5001 |
| # cluster 64 - Size: 1 |  |  |
|  | XP\_065067347\_5268-model64 | -72.3375 |
| # cluster 65 - Size: 1 |  |  |
|  | XP\_065067347\_5268-model65 | -72.2129 |
| # cluster 66 - Size: 1 |  |  |
|  | XP\_065067347\_5268-model66 | -71.8813 |
| # cluster 67 - Size: 1 |  |  |
|  | XP\_065067347\_5268-model67 | -71.6361 |
| # cluster 68 - Size: 1 |  |  |
|  | XP\_065067347\_5268-model68 | -71.5186 |
| # cluster 69 - Size: 1 |  |  |
|  | XP\_065067347\_5268-model69 | -71.4456 |
| # cluster 70 - Size: 1 |  |  |
|  | XP\_065067347\_5268-model70 | -70.9844 |
| # cluster 71 - Size: 1 |  |  |
|  | XP\_065067347\_5268-model71 | -70.8304 |
| # cluster 72 - Size: 1 |  |  |
|  | XP\_065067347\_5268-model72 | -70.828 |
| # cluster 73 - Size: 1 |  |  |
|  | XP\_065067347\_5268-model73 | -70.8199 |
| # cluster 74 - Size: 1 |  |  |
|  | XP\_065067347\_5268-model74 | -70.3346 |
| # cluster 75 - Size: 1 |  |  |
|  | XP\_065067347\_5268-model75 | -69.7023 |
| # cluster 76 - Size: 1 |  |  |
|  | XP\_065067347\_5268-model76 | -69.155 |
| # cluster 77 - Size: 1 |  |  |
|  | XP\_065067347\_5268-model77 | -69.1344 |
| # cluster 78 - Size: 1 |  |  |
|  | XP\_065067347\_5268-model78 | -68.9854 |
| # cluster 79 - Size: 1 |  |  |
|  | XP\_065067347\_5268-model79 | -68.7913 |
| # cluster 80 - Size: 1 |  |  |
|  | XP\_065067347\_5268-model80 | -68.3498 |
| # cluster 81 - Size: 1 |  |  |
|  | XP\_065067347\_5268-model81 | -68.1462 |
| # cluster 82 - Size: 1 |  |  |
|  | XP\_065067347\_5268-model82 | -67.6679 |
| # cluster 83 - Size: 1 |  |  |
|  | XP\_065067347\_5268-model83 | -67.4939 |
| # cluster 84 - Size: 1 |  |  |
|  | XP\_065067347\_5268-model84 | -67.3839 |
| # cluster 85 - Size: 1 |  |  |
|  | XP\_065067347\_5268-model85 | -67.1232 |
| # cluster 86 - Size: 1 |  |  |
|  | XP\_065067347\_5268-model86 | -66.7236 |
| # cluster 87 - Size: 1 |  |  |
|  | XP\_065067347\_5268-model87 | -65.3041 |
| # cluster 88 - Size: 1 |  |  |
|  | XP\_065067347\_5268-model88 | -64.7782 |
| # cluster 89 - Size: 1 |  |  |
|  | XP\_065067347\_5268-model89 | -64.3707 |
| # cluster 90 - Size: 1 |  |  |
|  | XP\_065067347\_5268-model90 | -63.0518 |
| # cluster 91 - Size: 1 |  |  |
|  | XP\_065067347\_5268-model91 | -62.4672 |
| # cluster 92 - Size: 1 |  |  |
|  | XP\_065067347\_5268-model92 | -61.3751 |
|  |  |  |
